# “*Skills for Resilience in Farming*”; an evidence-based, theory driven educational intervention to increase mental health literacy and help-seeking intentions among Irish farmers

**DOI:** 10.1371/journal.pone.0333115

**Published:** 2025-10-01

**Authors:** Siobhán O’Connor, Sandra M. Malone, Joseph Firnhaber, Sinéad O’Keeffe, John McNamara, Anna Donnla O’Hagan

**Affiliations:** 1 School of Health and Human Performance, Dublin City University, Glasnevin Campus, Dublin, Ireland; 2 Teagasc - Irish Agriculture and Food Development Authority, Farm Health and Safety Knowledge Transfer Unit, Kildalton, Piltown, Kilkenny, Ireland; University of Florida, UNITED STATES OF AMERICA

## Abstract

While mental health literacy is an important component to successful help-seeking, rural populations often face gaps in both knowledge and service provision. Informed by the Theory of Planned Behaviour and Self-Efficacy Theory, we designed the ‘Skills for Resilience’ as a brief, once-off, community-based educational intervention to increase Irish farmers’ mental health literacy and help-seeking intentions. We adopted a quasi-experimental between (group: intervention and control) and within-group design (time: baseline [T1], immediately post-intervention [T2], and ≥ 1 month post-intervention [T3]). A total of 72 participants (intervention n = 37; control n = 35) were recruited from knowledge-sharing discussion groups. Although recruitment was also open to women, all discussion groups consisted of men. A trained facilitator delivered a discussion lasting between 30 and 90 minutes. Five intervention participants also participated in a qualitative interview after T3. Our results identified intervention participants’ mental health literacy increased significantly at T2 and T3 compared to T1, but did not increase between T2 and T3. Mental health literacy was also significantly greater in the intervention group compared to the control group at T2 and T3. Help-seeking intentions and self-efficacy in seeking mental healthcare also increased significantly at T2 compared to T1, but did not increase between T1 and T3 or T2 and T3. There were no significant changes in outcome measures for the control group at any time point. Through reflexive thematic analysis we identified that the intervention also addressed stigma against mental health (Theme 1) and provided important resources for participants and their community’s present and future coping (Theme 2). At T3, 100% of participants enjoyed the discussion and would recommend the intervention to other farmers. This intervention provides a successful example of integrating the Theory of Planned Behaviour and Self-Efficacy Theory to improve mental health literacy in farmers using a brief, educational intervention.

## Introduction

Farmers globally can experience ill mental health at higher rates than the general population [1–3] and represent an important high-risk target group for preventative interventions [4]. Rates of suicide are significantly higher in younger (≤35 years) and older (≥55 years) farmers compared to non-farmers [5], while both suicide and depression rates are higher in farmers worldwide, than any other occupational group [6–8]. Working long hours in isolation, often without support, and contending with financial and regulatory pressures are likely to contribute to the experience of mental health issues [9–10]. In addition, the magnitude of uncertainty entailed in farming, such as changing regulations, disease outbreak and unpredictable weather, is a contributory factor to poor mental health and suicide [6,11]. In Ireland, preliminary findings from Russell et al. [12] found that from 2014 to 2020, Irish farmers experienced higher mortality rates from suicide than from farm accident fatalities, and attributed this to stressors such as succession, isolation, financial concerns, bureaucracy, and climate change. The farmer’s mental health affects not only the farmer alone but can also extend to family life [13], farm productivity [14] and animal welfare [15]. The high rates of poor mental health and suicide necessitates the urgent need for mental health interventions among farmers.

Despite facing significant mental health stressors, and the availability of mental health services (e.g., counselling, helplines), help-seeking is not common in farmers and uptake of mental health services is poor [16]. While residing in rural areas may increase the difficulty of accessing health services [17], cultural barriers such as stigma, self-reliance and masculine norms can also deter farmers from seeking help [17–19]. Farmers can often perceive help-seeking as a sign of weakness [6,16,20,21] and may instead rely on informal mental health help from family and friends rather than professional assistance [22–24]. While social support is critical to living with and recovering from mental illness [25], family and friends are often untrained in how to appropriately manage mental illness and support these individuals [26]. Mental health literacy, described as the knowledge of mental health disorders and the ways to support and reduce them [27], is associated with help-seeking [28–29]. Irish farmers have low rates of both mental health literacy and help-seeking [30]; if farmers are unaware of the signs of poor mental health, they may not realise that they are struggling and need help.

Enhancing the resilience and self-efficacy needed to manage and overcome uncertainty as well as to increase help-seeking intentions [31] may be the key to addressing the rates of mental health issues in farmers. Self-efficacy, the belief one has in their ability to execute a behaviour and attain the desired outcome [32], is inversely related to anxiety and depression [33–34], but more importantly, is one of the most significant predictors of adopting and maintaining health behaviours [35]. Human functioning is facilitated by a sense of perceived control (self-efficacy), and so, if an individual feels that they can solve problems, they become more motivated and committed to doing so [36]. Self-efficacy is a strong determinant of coping in the face of adversity and may be a protective factor for poor emotional responses [37]. Furthermore, self-efficacy can help people bridge the gap between help-seeking intentions and action [36,38,39] and is also a predictor of help-seeking behaviours in rural populations [17]. The Theory of Planned Behaviour (TPB) is also a useful framework for understanding the factors that influence formal help-seeking [40–41] and has been successfully applied in many interventions to increase help-seeking behaviour [42–43]. TPB suggests that intention is key to behaviour change [40] and is determined by attitude (perceived consequences of behaviour change), subjective norms (perceived expectation of others) and perceived behavioural control (barriers or facilitators to behaviour change) [40,42,44]. By increasing self-efficacy (internal perceived behavioural control), intentions to seek help can potentially be increased [45].

Resilience, the mental processes (i.e., problem solving) and protective behaviours (i.e., accessing support) from stressors [46], has been widely documented as a buffer against adverse outcomes, such as depression, anxiety, and stress [47–48]. In farming, resilience emphasises the importance of persistence to enable coping and adaptation to changing regulations, or adverse situations e (e.g., bad weather) [49]. A combination of resilience, self-efficacy and mental health literacy may be key to ensuring optimal mental health outcomes in farmers when they encounter stressors.

Promoting mental health and wellbeing among farmers through education can strengthen personal resources, such as resilience and self-efficacy, needed to endure the challenges in farming. Mental health literacy programmes in Australia [50] and New Zealand [51] have successfully increased mental health knowledge, health promoting behaviours and improved attitudes towards mental health in farmers [52]. To the best of the authors’ knowledge, no farmer-specific intervention has been developed to directly address poor mental health literacy and help-seeking intentions evident in Irish farmers. Educational interventions are the most effective when utilising a theory-based approach [53] and considering end-users’ recommendations for content and design [54]. Incorporating both service users and providers enhances credibility in the intervention [55] and is likely to result in successful implementation [56]. Therefore, the current study aimed to develop and examine the effectiveness of a bespoke evidence-based educational intervention to increase help-seeking intentions, mental health literacy, self-efficacy, and knowledge of mental health promoting behaviours among Irish farmers.

## Materials and methods

### Research design

**Fig 1:**
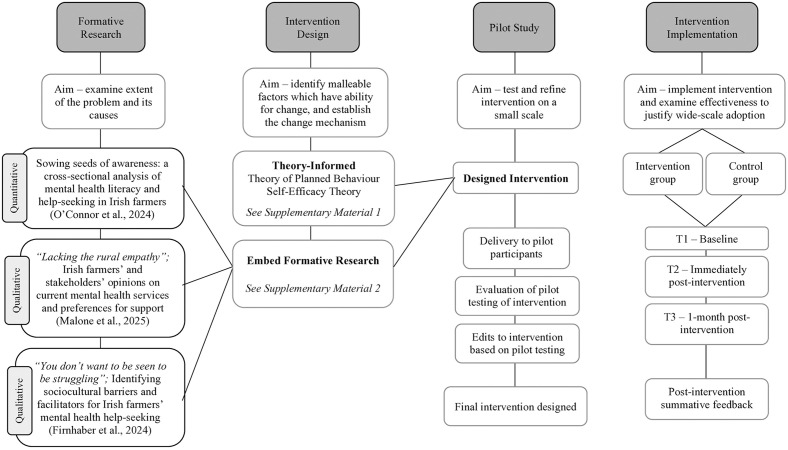
Logic map representing design of the research.

In the development of a public health intervention, the six steps for quality intervention development outline the need to 1) examine the extent of the problem and its causes, 2) identify which factors are malleable and have the ability for change, 3) identify the change mechanism and 4) how to deliver that change, 5) test and refine on a small scale and 6) implement intervention and examine effectiveness to justify wide-scale adoption [57]. This processed was adhered to in the current study and the logic map representing the design of the research is evident in Fig 1.

### Intervention design

We developed the ‘*Skills for Resilience*’ as a brief, once-off, community-based educational intervention to increase Irish farmers’ mental health literacy and help-seeking intentions according to the preferences for mental health supports of Irish farmers and farming stakeholders [58]. The intervention was underpinned by the TPB [40] and Self-Efficacy Theory [32,59] (S1 File). A detailed description of the challenges to engaging farmers in mental health interventions and how we addressed them, is contained in S2 File.

The “*Skills for Resilience*” educational intervention was designed by the research team using their expertise in mental health and their farming backgrounds and was informed by similar short intervention programs designed to improve mental health literacy in sport [60–61]. The intervention included content on signs of poor mental health and associated factors (stress, sleep issues, burnout), positive mental health behaviours (nutrition, sleep routine, hobbies, time off), recognition of their own and others’ mental distress and how to access available mental health resources. The content was designed to be delivered in-person through a once-off discussion (S3 File), supplemented by a laminated A5-sized handout with mental health and farm supports, and a website (farmhealth.wixsite.com/farmhealth). The website contained five videos on 1) signs a farmer may be struggling with their mental health 2) strategies for resilience, 3) mental health and farming supports, 4) and 5) two Irish farmers separately describing their own mental health challenges and how they sought help.

### Pilot study

The intervention was piloted with 5 farmers in one discussion group (34.2 ± 15.0 years; male *n* = 3 and female *n* = 2; dairy *n* = 4, dairy and beef *n* = 1). Verbal evaluation and feedback obtained post-intervention demonstrated the pilot study was well received and accepted by participants. Following pilot feedback, the content of the talk was reduced to ensure the discussions would not be excessively long in duration and additional farming examples were included.

### Intervention implementation

#### Design.

This study used a parallel mixed-methods approach, which allowed for a more thorough investigation of the intervention efficacy [62]. A quasi-experimental design, with a between (group: intervention and control) and within-group design (time: baseline [T1], immediately post-intervention [T2], and ≥ 1-month post-intervention [T3]) was implemented. Qualitative feedback was collected at T3 and in post-intervention interviews. Data was collected between September 2023 to January 2024.

#### Participants.

Irish farmers (*n* = 72; age = 49.9 ± 11.4 years) were recruited to an intervention group (*n* = 37; age = 48.1 ± 12.4 years) or control group (*n* = 35; age = 51.7 ± 10.1 years). All participants in this study were male. Farmers were predominantly dairy (*n* = 28, 39%), followed by dairy and beef (*n* = 17, 24%), beef (*n* = 15, 21%), sheep and beef (*n* = 11, 15%) and sheep and tillage (*n* = 1, 1%). The sample is representative of the overall Irish farming population as the majority of Irish farmers are male (87%), aged 45–64 (46.6%) and dairy and/or beef farmers (66.3%) [63].

#### Procedures.

Ethical approval was obtained from the Institutional Research Ethics Committee (#2023/122) and informed online consent was provided prior to participation. A sample size calculation using G*Power [64] indicated that at least 44 participants (22 in the intervention group and 22 in the control group) were required, with an alpha level of 0.05, effect size of 0.25, power of 0.95, two groups, and three time points. After adjusting for a 20% dropout, a minimum of 54 participants (27 in each group) were required.

Based on farmers’ preferences for a mental health intervention [58], the intervention was delivered as a once-off discussion through farmer discussion groups, which were recruited from counties across the Irish midlands through Teagasc, the state Agriculture and Food Development Authority. Discussion groups consist of 10–15 local farmers that meet monthly on one of the group members’ farms, facilitated by a trained Teagasc advisor. Discussion groups provide an opportunity to share ideas and solve problems, and also have a social aspect, where farmers can find support and create new friendships [65–66]. Once permission was granted from each discussion group’s chairman and advisor to attend their group, each discussion group was randomly assigned to either the intervention or control group. Intervention and control groups completed previously developed valid and reliable measures of help-seeking intentions, mental health supports, help-seeking self-efficacy and knowledge of factors promoting good mental health (Table 1) at T1, T2 and T3. The intervention was facilitated by a qualified psychologist with master’s qualifications and a farming background (member of the research team). The intervention was provided to the control group, after completing all measures, to ensure that they also received this important educational intervention, without impacting their status as a control group. Follow-up was completed either in-person or online on Qualtrics from 3–12 weeks (8.38 ± 1.89 weeks) post-intervention. Discussions ranged from 30–90 minutes in duration (62.5 ± 32.02).

**Table 1:** Measures and scoring system used in the “Skills for Resilience” intervention questionnaires.

		Scoring		
Measure	No. of items	Likert Rating System*	Total score range*	Score Interpretation
Mental Help Seeking Intention Scale [67]	3	7-point scale: 1 (*extremely unlikely*) to 7 (*extremely likely*)	1-7 (mean score used)	Higher score = higher help-seeking intention
Multicomponent Mental Health Literacy- Resource oriented subscale [68]	4	2-point scale: 0 (*no*) to 1 (*yes*)	0-4 (total score used)	Higher score = greater knowledge of mental health supports
Self-Efficacy in Seeking Mental Health Care Scale [69]	9	10-point scale: 1 (*no confidence*) to 10 (*complete confidence*)	9-90 (total score used)	Higher score = higher self-efficacy in executing each behaviour listed
Mental Health Promoting Knowledge [70]	10	5-point scale: 1 (*completely wrong)* to 5 (*completely correct),* with extreme value option (*don’t know)*	0-5 (mean score used; *don’t know* scored as 0)	<4 = insufficient knowledge of positive mental health habits

*Rating systems and scoring (total score/mean score) are those originally developed by the authors of each measure

Five farmers (42.2 ± 11.7 years) completed a semi-structured interview 11–15 weeks post-intervention, on Zoom (version 5.17.1) to assess perceptions of the 1) “*Skills for Resilience*” educational discussion and 2) supplementary material. The interview guide (S4 File) was developed and refined using the authors’ farming/ rural background and mental health expertise. Interviews were video recorded on Zoom and transcribed verbatim. To preserve anonymity and confidentiality, participants were assigned identification numbers during transcription (e.g., P1 = participant 1). The average interview duration was 27.8 ± 16.72 minutes.

#### Measures.

##### Mental Health Help-Seeking Intention Scale (MHSIS)

MHSIS (Table 1) is a self-report measure designed to assess intentions to seek help from a mental health professional if having a mental health concern. The MHSIS has demonstrated reliability when employed with people experiencing mental health issues (α = .94) [67] and the general population (α = .97) [71]. Good internal consistency was shown in the current sample at T1 (α = .93), T2 (α = .98) and T3 (α = .93).

##### Multicomponent Mental Health Literacy Scale- Resource Oriented Subscale (MMHL-RO)

The MMHL scale is designed to determine the mental health literacy of lay community members and has demonstrated good internal consistency when used accordingly (α = .84) [68]. We used the resource-oriented subscale of MMHL (items 23–26) (Table 1), designed to assess people’s knowledge of available mental health supports and information. Good internal consistency of MMHL-RO subscale was shown in the current sample at T1 (α = .81), T2 (α = .85) and T3 (α = .81).

##### Self-Efficacy in Seeking Mental Healthcare Scale (SE-SMHC)

SE-SMHC scale (Table 1) assesses self-efficacy in seeking health care for mental health, specifically the confidence in one’s ability to cope with the consequences of seeking care and the confidence in one’s ability to know how to successfully interact with mental health care systems [69]. The SE-SMHC scale has shown good internal consistency (α = .93) and validity in the general population [69]. Good internal consistency was evident at T1 (α = .93), T2 (α = .95) and T3 (α = .95).

##### Mental Health Promoting Knowledge Scale (MHPK)

The MHPK scale (Table 1) is used to assess knowledge of behaviours important to positive mental health. MHPK demonstrates good reliability when employed with adolescents (α = 0.74) [70]. Good internal consistency of MHPK was shown in the current sample at T1 (α = .83), T2 (α = .93) and T3 (α = .78).

### Data analysis

#### Quantitative.

Data were analysed using IBM SPSS Statistics (version 28.0.1.1). An alpha level of 0.05 was used for all tests. Descriptive statistics were used to analyse demographics (age, farm type). Separate independent samples t-tests (age and measures at T1) or Fisher exact tests (main farm type) were used to examine differences between intervention and control groups. Effect sizes were interpreted using Cohen’s *d* and classified as small (*d* = 0.2), medium (*d* = 0.5) and large (*d* = 0.8) [72]. Linear Mixed Modelling (LMM) was used to examine differences between groups over time for each of the dependent variables (MHSIS, MMHL-RO, SE-SMHC and MHPK). LMM was employed as it allows for analysis of data with missing values at different time points, unequal variances and correlated data that is common with repeated measures [73]. We conducted LMM with group, time and group-by-time interaction treated as fixed effects and participants as a random effect. Bonferroni adjustments for multiple comparisons were applied to limit type I error.

#### Qualitative.

Qualitative feedback on the intervention was collected at the end of the T3 survey and in post-intervention interviews. Interviews were transcribed verbatim, and all qualitative data was coded manually and using NVivo (QSR International Pty Ltd.). We analysed participants’ coded responses using reflexive thematic analysis [74–75]. Through participants’ experiences, we aimed to identify the intervention’s subjective impacts, including and beyond our expected outcomes measured by our quantitative analysis. We utilised the “critical friend approach” [76–77] to maintain rigour, and developed themes collaboratively and through continually challenging our interpretation. Extracts presented in our final analysis were chosen collaboratively to best represent participants’ accounts of participating in our intervention.

## Results

**Fig 2:**
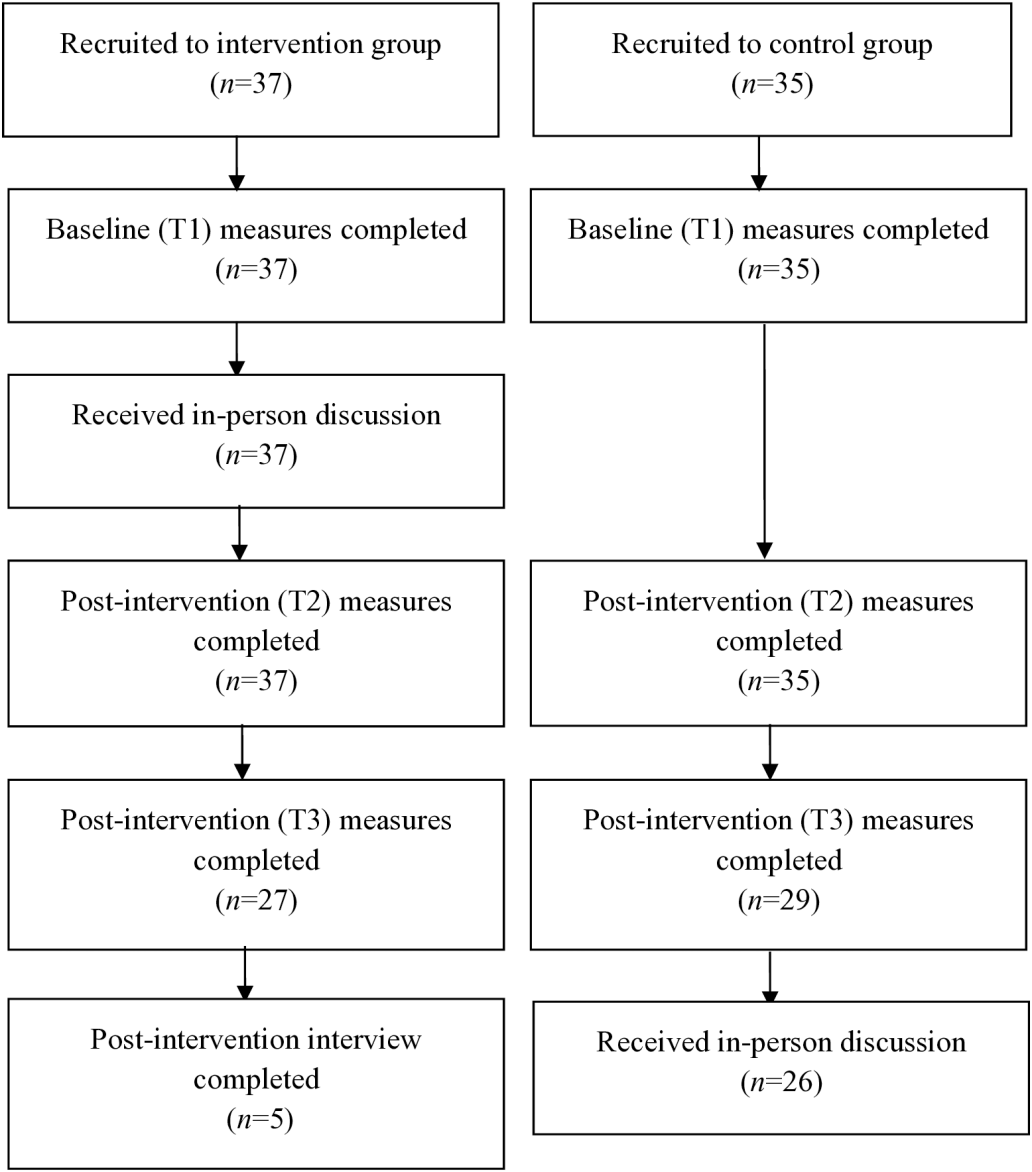
Flow chart of participants in study.

Of the 37 participants recruited to the intervention group, 27 (73%) completed the follow-up at T3, whereas in the control group (*n* = 35), 29 (83%) completed the T3 follow-up (Fig 2). No differences were evident between groups for age (*p* = .19), but a significant difference was noted for main farm type (*p* < .001; *d* = .53) (Table 2). Mean scores in outcome measures at baseline and post-intervention are presented in Table 3.

**Table 2:** Participant demographic information.

	Group									
	Intervention					Control				*P* Value
	1	2	3	4	Total	1	2	3	Total	
	(n = 10)	(n = 11)	(n = 8)	(n = 8)	(n = 37)	(n = 11)	(n = 15)	(n = 9)	(n = 35)	
**Main Farm Type** **n (%)**										<.001
Dairy	–	4 (36.4)	5 (62.5)	7 (87.5)	16 (43.3)	10 (90.9)	–	2 (22.2)	12 (34.3)	
Beef	–	–	1 (12.5)	–	1 (2.7)	–	14 (93.3)	–	14 (40.0)	
Dairy and beef	–	6 (54.5)	2 (25.0)	1 (12.5)	9 (24.3)	1 (9.1)	–	7 (77.8)	8 (22.9)	
Sheep and beef	9 (90.0)	1 (9.1)	–		10 (27.0)	–	1 (6.7)	–	1 (2.8)	
Sheep and tillage	1 (10.0)	–	–		1 (2.7)	–	–	–	–	
**Age** (years)	54.1 ± 10.6	44.1 ± 10.9	48.4 ± 14.2	45.9 ± 13.9	48.1 ± 12.4	48.3 ± 9.7	55.7 ± 11.5	49.1 ± 5.7	51.7 ± 10.1	.19

**Table 3:** Outcome measures.

		Mean ± Standard Deviation		
Outcome Measure	Group	T1^a^	T2^a^	T3^a^
Mental Health Help-Seeking Intention	Intervention Control	5.0 ± 1.7 5.3 ± 1.7	5.9 ± 1.3 5.6 ± 1.7	5.2 ± 1.4 5.4 ± 1.5
Multicomponent Mental Health Literacy (Resource oriented subscale)	Intervention Control	2.7 ± 1.4 2.5 ± 1.6	3.4 ± 1.1 2.6 ± 1.6	3.7 ± 0.7 2.9 ± 1.5
Self-Efficacy in Seeking Mental Health Care	Intervention Control	64.2 ± 15.9 62.7 ± 20.1	73.4 ± 15.0 67.7 ± 19.6	67.7 ± 16.1 66.3 ± 19.0
Mental Health Promoting Knowledge	Intervention Control	4.4 ± 0.5 4.3 ± 0.7	4.4 ± 0.9 4.4 ± 0.7	4.3 ± 0.6 4.5 ± 0.5

^*a*^ *T1, baseline; T2, immediately post-intervention; T3, ≥ 3 weeks post-intervention*

### Outcome measures

#### Mental health help-seeking intention scale.

**Fig 3:**
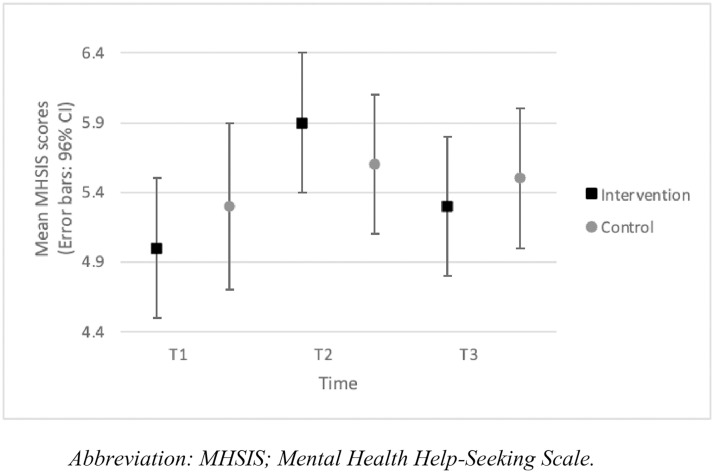
MHSIS scores per group across the intervention (mean, 95% CI).

A significant group-by-time interaction was evident for MHSIS (*F*_(2,66.6)_=3.5; *p* = .04). In the intervention group, the MHSIS score significantly increased from T1 to T2 (*p* < .001; *d* = 0.6) but was not significantly different between T1 and T3 or T2 and T3 (*p* > .05). This shows mental health help-seeking intentions improved after participation in the educational intervention, but these changes were not sustained at follow-up. We observed no significant differences in the control group between T1, T2 and T3 (*p* > .05). No significant differences were evident in MHSIS scores between the intervention and control groups at T1, T2 and T3 (*p* > .05) (Fig 3).

#### Multicomponent mental health literacy (Resource-Oriented Subscale).

**Fig 4:**
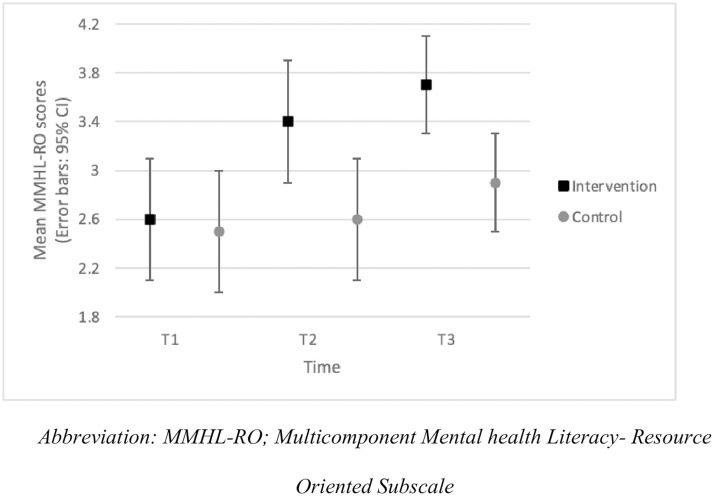
MMHL-RO subscale scores per group across the intervention (mean, 95% CI).

We observed a significant group-by-time interaction for MMHL-RO (*F*_(2,65.8)_=3.9; *p* = .03). In the intervention group, the MMHL-RO score significantly increased from T1 to T2 (*p* < .001; *d* = 0.6) and T1 to T3 (*p* < .001; *d* = 1.0). The scores were not different between T2 and T3 (*p* > .05). These findings show mental health literacy, particularly knowledge of mental health supports, improved as a result of participation in the educational intervention, and those changes were sustained at follow-up. No significant differences were observed in the control group between T1, T2 and T3 (*p* > .05). We noted no significant differences in MMHL-RO scores between intervention and control groups at T1 (*p* = .71), but differences were evident at T2 (*p* = .01; *d* = 0.6) and T3 (*p* = .01; *d* = 0.7) (Fig 4).

#### Self-Efficacy in seeking mental health care.

**Fig 5:**
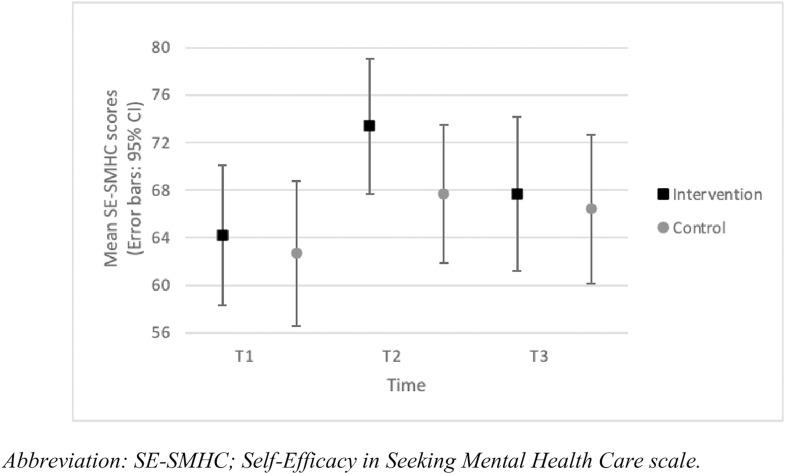
SE-SMHC scores per group across the intervention (mean, 95% CI).

We observed no significant group-by-time interaction for SE-SMHC (*F*_(2,63.5)_=1.2; *p* = .30). In the intervention group, SE-SMHC significantly increased from T1 to T2 (*p* < .001; *d* = 0.6) but was not significantly different between T1 and T3 or T2 and T3 (*p* > .05), which shows self-efficacy in seeking health care for mental health improved for those that participated in the educational intervention, but those results were not sustained at follow-up. No differences were observed in the control group between T1, T2 and T3 (*p* > .05). We noted no significant differences in SE-SMHC between the intervention and control groups at T1, T2 and T3 (*p* > .05) (Fig 5).

#### Mental health promoting knowledge scale.

**Fig 6:**
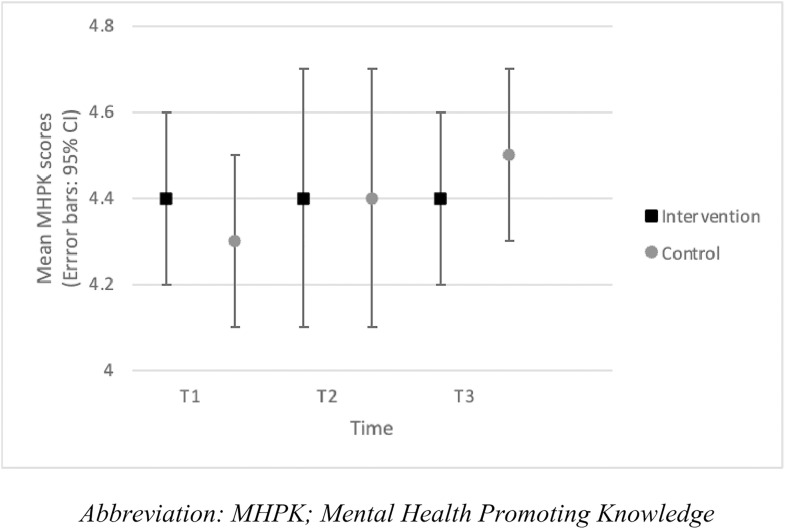
MHPK scores per group across the intervention (mean, 95% CI).

We observed no significant group-by-time interaction for MHPK (*F*_(2,65.9)_=1.6; *p* = .22). No significant differences were evident in the intervention or control group between T1, T2 and T3 (*p* > .05). These findings show, regardless of participation in the educational intervention, knowledge of positive mental health habits did not significantly change. We noted no significant differences in MHPK scores between the intervention and control groups at T1, T2 and T3 (*p* > .05) (Fig 6).

### Participant feedback

Of the participants that completed the survey at T3 (*n* = 27, 73%), 100% indicated that they enjoyed the ‘mental health discussion’. In addition, 100% of the T3 follow-up participants (*n* = 27) said that they would recommend the programme to other farmers. A small percentage of intervention participants (30%; *n* = 8) reported engagement with the online supplementary website. Additional participant responses from open-ended feedback questions and interviews are described below.

### Qualitative analysis

We aimed to identify participants’ experiences of participating in, and opinions about the effectiveness of, the intervention. Through reflexive thematic analysis of participants’ interviews and answers to our open-ended survey questions, we identified two central themes. Across both themes, participants spoke about the effectiveness of the intervention, with their main critiques being its limited scope. In Theme 1, *Challenging Stigma*, participants described how creating a context for the open discussion of mental health concerns was one of the most powerful impacts of the intervention, and likely reduced mental health stigma. In Theme 2, *Providing Resources*, participants described how the intervention materials were effective at providing a list of coping strategies and resources for farmers to support their own and others’ mental health, even when farmers were already aware of these strategies or resources.

#### Theme 1: Challenging stigma.

In Theme 1, the majority of participants described that an important impact of this intervention was addressing the widespread stigma against mental illness by providing a rare context for discussion of mental health issues. Most participants identified how important reducing stigma is in an Irish rural context. P1 and P3 provided the most direct claims.

*“Well just that it wasn’t stigmatised. I suppose [the discussion group] was a place that you could talk about it because, like I’ve never really been at an event that you could talk about mental health” (P1).*

“*With the negativity of the subject [...] stigma and all that [...] the fact that you’re talking about it, you’re reducing the stigma. Which is nearly more important than looking for the signs [and] the symptoms” (P3).*

Like P3, participants welcomed the discussion on mental health, with many expressing relief that the topic was broached despite the surrounding stigma. For example, P1 said that *“What you’re doing is reducing all that stigma and shame and that’s very important.”* Participants’ open-ended answers echoed this sentiment, as they shared that it was “*great to talk about what is considered such a taboo subject,*” and that the discussion was able to “*increase awareness of a huge problem*”. Put otherwise, participants reported that a possible outcome of our intervention was not only increased mental health awareness, but a reduction in participants’ perceived stigma against mental illness, and possibly a reduction in internalised stigma as well. Participants identified how both the facilitator and the context of this intervention were important for enabling this discussion:

“*[The facilitator] made it very easy to discuss mental health and the challenges faced by farmers. I learned a lot at the discussion and enjoyed talking to someone that understands both farming and mental health, it was fantastic*” (P5).

“*Discussion groups are a great place for the mental health discussion. Lads know each other and feel comfortable, and a lot of problems are solved there. Great social aspect to the group and having the mental health discussion there is the way forward*” (P1).

Across their responses, many participants described feeling comfortable with both the facilitator and others involved in the discussion, and therefore being able to engage in discussions that collectively raised awareness instead of contributing to stigma about this sometimes ‘*taboo*’ topic. Indeed, the only improvements participants suggested regarding the intervention’s handling of stigma and awareness were expansions rather than modifications to the program, specifically to more isolated farmers. As P2 described, “*a lot of farmers I know wouldn’t actually be into the discussion groups but if you had a little trailer out the side of the marts near the canteen you might get them talking. It’s so isolated, the farming*.” However, participants also warned that an expansion into marts or similar contexts would have different obstacles to overcome and would lack the easy engagement and emotional intimacy of discussion groups. As P4 cautioned, *“it’s hard to see lads cooperating at a mart. If it was 10 random farmers at table, I’d say it’d be a lot different.”*

#### Theme 2: Providing resources.

In Theme 2, many participants described how the other important impact of participating in this intervention was learning about more resources they could draw on to support their own and other’s coping. Though, unlike stigma, participants did not name mental health literacy explicitly, many of their descriptions of coping resources match elements of mental health literacy, especially mental illness management. Participants notably stressed the importance of applying their knowledge to help others in their communities manage their mental health and illness, as P4 described:

“*It’d be a lot easier to say something to someone now than it would have before. Cause I didn’t know how to deal, I didn’t know what to say, or even where to point them to go in the right direction.*”

It is entirely possible that participants applied this knowledge to others rather than themselves to sidestep facing any of the pervasive stigma identified in Theme 1. However, participants also expressed great willingness to care for and express solidarity with their communities, as P2 described, “*Yeah, I’d have no issues [now] talking about [mental health] to anyone or if anyone needed an open [chat] - or just needed to be heard. [...] We all are going through the same.”* Participants echoed these views in their open-ended answers, saying that the intervention helped them “*find out how to spot the signs and how to get help for ourselves and others”* and that “*we all suffer from stress and worries at times and it’s a great help to reach out.”* Even farmers who described that the mental health knowledge was not immediately applicable described its value, as P5 described through a clever analogy:

“*Anything like that is never any harm. I had the truck licence, and I said, I don’t know, will I ever use it? [Someone] said “It’s no load to carry, you know, the fact that you have it [is enough.]” And so, it’s like that’s the way I was thinking about these conversations, you put them in the toolbox at the back of your brain somewhere and maybe sometime, you will need it.*”

By far, many interview participants identified that the most useful materials in supporting their coping were the physical handout and the video testimonials. Participants described these as “*excellent*” and “*thorough*,” and similar to Theme 1, their main criticisms were the limited scope of the initiative. For example, participants found the video testimonials especially relatable, and requested “*more case studies*” and “*more personal stories*.” While participants noted that some farmers would likely ignore or discard the physical handout, P4 argued that, to compensate, the handout “*should be everywhere. Everyone should have one of them. [...] The department [Irish department of agriculture] should be sending out that one with letters.*” Essentially, farmers said they learned much more from and had a clear preference for materials (like the handout and testimonial videos) to be presented in person at the discussion groups rather than designed to be viewed in their own time after the discussion.

## Discussion

We aimed to develop, deliver, and examine the effectiveness of ‘*Skills for Resilience,*’ a brief, once-off, community-based educational intervention to increase Irish farmers’ mental health literacy and help-seeking intentions. We identified two central impacts of this initiative. First, the intervention was successful in its primary objective; participants showed significant, lasting improvements in mental health literacy, and short-term improvements in both help-seeking intentions and self-efficacy. Second, as identified across two qualitative themes, the intervention addressed stigma and provided important resources for participants’ present and future coping. In Theme 1, the intervention was successful in counteracting stigma against mental health issues and help-seeking in a comfortable environment, and as described in both Theme 2 and the analysis of participant’s feedback, the context and content of the intervention was universally well received and deemed useful by participants, with the only negative feedback being the limited scope. In balance, the intervention did not produce significant results for mental health promoting knowledge, and the short-term improvements in help-seeking intentions and self-efficacy were not significantly different from the control.

### Impact 1: Increased mental health literacy

The primary impact of this intervention was to make lasting improvements to Irish farmers’ mental health literacy, specifically their knowledge of and ability to access mental health resources, at both T2 and T3. Even more, while the intervention (2.7 ± 1.4) and control (2.5 ± 1.6) groups showed no significant difference in their mental health literacy scores at T1, participants in the intervention group reported significantly higher mental health literacy immediately after participating in the intervention (T2 = 3.4 ± 1.1; p < .001; d = 0.6) and at follow-up (T3 = 3.7 ± 0.7; p < .001; d = 1.0) when compared to those in the control group, who reported no significant changes across time points (p > .05). This significant impact is especially noteworthy considering the lack of mental health knowledge, specifically how to access mental health resources, and stoic attitudes towards mental illness described by some Irish farmers [19] and well documented in farmers internationally [4]. Therefore, our results further illustrate the suitability of MHL education for populations with comparatively low MHL, adding to positive results with farmers in Canada [52] and athletes in Ireland [61]. Even more, our results further illustrate that even a once-off, brief discussion of MHL can have a substantial effect. This efficiency is especially pragmatic for interventions trying to overcome common barriers in providing mental health service to farmers, including unpredictable schedules, geographical isolation, and tight time constraints [6,78]. Finally, this result expands on literature linking MHL to resilience [79] by effectively framing skills related to mental health literacy, such as symptom recognition, as an essential component of resilience. As our use of resilience was recommended by the Irish farming community as a desirable value for farmers [58], we recommend that future MHL interventions, especially catered to populations with low MHL or that value stoicism, work with and incorporate service users existing understanding of mental health and illness rather than directly challenging their beliefs.

The ‘*Skills for Resilience*’ intervention also produced short-term improvements in farmers’ help-seeking intentions and self-efficacy. Specifically, intervention participants reported significantly higher mental health help-seeking intentions (T1 = 5.0 ± 1.7; T2 = 5.9 ± 1.3; T3 = 5.2 ± 1.4) and self-efficacy in seeking mental health care (T1 = 64.2 ± 15.9; T2 = 73.4 ± 15.0; T3 = 67.7 ± 16.1) immediately after the intervention (T2; MHSIS: p < .001; d = 0.6; SE-SMHC: p < .001; d = 0.6), but not at follow-up (T3; p > .05). This finding illustrates the importance of structuring MHL interventions around TPB principles, as they can meaningfully impact participants’ self-efficacy and behavioural intentions [80]. The non-significant difference between the intervention and control groups’ help-seeking intention and self-efficacy scores might suggest that many participants were already amenable to seeking mental health help. Indeed, farmers who sought help in the past were more likely to seek help again [16]; future intervention studies could measure past-help-seeking behaviour or history of mental illness to control for this. For example, in their open-ended responses, participants described that the intervention was a good reminder of the importance of seeking help or that it was good that someone was ‘finally’ addressing the issue. Effectively, similar interventions may be more impactful for cohorts of farmers less amenable to help-seeking than our participants were. Together, these results add to a nuanced understanding of rural mental health literacy. Despite low levels of mental health literacy, farming communities not only recognise and appreciate the importance of mental health initiatives in their communities [58,81], but are also receptive to interventions which target mental health literacy [50–52].

### Impact 2: Challenging stigma, providing resources

This intervention was not explicitly designed to challenge negative mental health help-seeking stigma beyond addressing it as an important component of mental health literacy [27] and as an avenue of changing subjective norms around mental health [40]. Specifically, this intervention was designed to circumvent the stigma described by the Irish farming community as a barrier to both mental health literacy and help-seeking [19] and widely observed in other rural populations [17–18]. However, in Theme 1: *Challenging Stigma*, participants consistently identified that simply discussing mental health and help-seeking in a farming space is itself enough to challenge mental health stigma. Specifically, participants identified the existing peer relationships in discussion groups as well as the facilitator’s farming background as structural factors that enabled stigma reduction. This corroborates previous work identifying facilitated discussion groups as a valuable context for discussing farmers’ health [82–83] and expands the potential benefits to stigma reduction. These findings also provide an example for how elements of the informal and community-based help-seeking that farmers often rely on [16,18,23] can successfully be incorporated in more formal educational interventions. Effectively, by utilising a facilitator with a farming background and engaging farmers’ existing peer networks in open discussion of mental health, this intervention could shift or at least challenge subjective norms that mental health and help-seeking are ‘*taboo*’ subjects in farming.

Even more, this open discussion of mental health in a farming context was so effective that 100% of participants who provided feedback identified it as enjoyable and would recommend it to other farmers. In further support of contact models of counteracting stigma [84–85], Theme 2: *Providing Resources* highlighted the importance of including farmer’s testimonials of coping with mental illness through help-seeking alongside other adaptation strategies as one of the most impactful components of the intervention. In addition, participants described how they found the coping and help-seeking resources important even if they didn’t need to utilise them immediately; they recognised their value in supporting others in the farming community. Effectively, participants were able to understand mental health knowledge, including possible avenues of help-seeking as a protective and preventive component of resilience [47–48], as one of many ‘*tools in the toolbox*.’ This finding further supports evidence that providing participants with mental health skills stands to benefit individuals and communities alike [86]. Effectively, this intervention’s materials likely challenged stigma through blending contact (testimonials) and knowledge-based (help-seeking information) models [87]. We strongly recommend similar MHL initiatives explicitly measure their impact on participants’ perceived and internalised stigma.

### Pragmatics and considerations

These findings should be considered within the context of several important considerations related to conducting this intervention entirely through discussion groups as well as the supplementary material. First, due to the heterogeneous nature of discussion groups, our control group included farmers of different farm types, specifically more beef farmers, compared to our intervention. Similarly, although women were not excluded on purpose, the discussion groups recruited for this intervention consisted entirely of farming men. This is an important limitation to the generalisability of our findings, as women are more likely to seek mental health support than men, and their experiences with stress and other mental health concerns in farming are distinctly different than men [52,88]. Our rollout across discussion groups also restricted our participant pool in other ways, as discussion groups have diverse leadership, and many farmers are not involved in discussion groups at all. For example, the facilitator worked to recruit members of private consultant discussion groups to expand our participant pool from those involved with research and advisory board Teagasc, but this was not possible. Even more, as many farmers do not participate in discussion groups, our sample consists of farmers already committed to the close and often long-standing social connections that discussion groups often entail. As participants noted, this comfortable context made discussing a stigmatised topic of mental health much easier, but by definition excluded some of the farmers who may be in more dire need of mental health literacy education. Future work could, as our participants recommended, take place at farming community events such as farming marts to target those more socially and geographically isolated populations [89–90]. However, these interventions may also miss the closer and more confidential social environment of discussion groups [82–83].

Our results, especially our T3 results, should be considered in light of three important pragmatic limitations. While discussion groups provided an excellent setting for discussing mental health, they provided important logistical constraints that future farmer mental health initiatives should consider, especially when conducting longitudinal research. First, as farmers’ schedules are unpredictable, follow-up (T3) data collection was threatened by cancellations and rescheduling until relocated online. Second, the heterogeneity and sometimes flexible structure of discussion groups resulted in vastly differing levels of engagement. For example, while we designed discussions to be brief, some highly involved groups participated for up to 90 minutes due to the depth of discussion and willingness to engage. Similarly, differing levels of engagement led to dropout in the T3 pool. Third, between T2 and T3, participants were instructed to engage with supplementary material online, including the website and farmer testimonials. While we identified that only 30% of participants engaged with the website, the farmer testimonials were one of the most praised components of our intervention, one garnering over 1500 views (identified through website analytics). This uneven response shows farmers’ clear preference for information not only catered for farmers, but by farmers [91], and future initiatives should integrate service users’ voices as much as possible not only through collaboration in designing the intervention, but as a core part of the main intervention itself. For example, testimonials have been used to great success in promoting veteran’s help-seeking [92].

## Conclusions

We designed the ‘*Skills for Resilience*’ intervention as a once-off, facilitated discussion on mental health literacy supported by video testimonials of farmers’ mental health help-seeking experiences as well as physical and digital lists of mental health resources for Irish farmers. This intervention was significantly effective at increasing participants’ mental health literacy, with sustained differences between intervention and control groups identifiable at follow-up. This intervention also significantly increased participants’ help-seeking intentions and self-efficacy immediately following the intervention. Additionally, through qualitative analysis of participants’ experiences taking part, we identified two themes: *Challenging Stigma*, and *Providing Resource*s. Participants highlighted the importance of the discussion format in addressing stigma against mental illness and help-seeking alike and identified the importance of supplementary materials in encouraging effective and preventative coping for farmers and their communities.

Together, these findings provide further evidence that the TPB remains an excellent foundation for help-seeking interventions, with our participants reporting shifted subjective norms around mental health help-seeking including reduced perceived stigma. This intervention’s success further provides strong evidence for tailoring mental health literacy interventions to the specific linguistic and cultural context of service users; in this case framing components of mental health literacy, such as help-seeking avenues and symptom recognition (both for farmers and others in their communities), as important aspects of resilience. Indeed, considering farmers’ nearly universally positive feedback about both the content and context of this intervention, our results indicate how much farmers can engage with, rate positively, and enjoy participating in mental health initiatives when using a farmer-centric and community-based approach [93]. We therefore recommend that initiatives for farmer’s mental health work in collaboration with service users [94] to ensure that messaging is suitable to the cultural context of communities and can challenge rather than reinforce stigma [95]. We recommend that future interventions for farmers and other communities which face low mental health literacy involve service users in much of the design and delivery to promote engagement and ensure not only community-centred but community-led service provision.

## Supporting information

S1 File Incorporation of Theory of Planned Behaviour and Self- Efficacy Theory in the Intervention. (DOCX)

S2 File Overcoming barriers reported by the Irish farming community. (DOCX)

S3 File Final Intervention Script. (DOCX)

S4 File Semi-Structured interview Guide. (DOCX)
