# Supplementary material for: “Skills for Resilience in Farming”; an evidence-based, theory driven educational intervention to increase mental health literacy and help-seeking intentions among Irish farmers
Source: PLoS One. 2025 Oct 1;20(10):e0333115. doi: 10.1371/journal.pone.0333115 (PMC12488010; doi:10.1371/journal.pone.0333115)
Supplement: S1 File — (DOCX) [file pone.0333115.s001.docx]

**Supplementary Material 1**

*Incorporation of Theory of Planned Behaviour and Self- Efficacy Theory in the Intervention*

We developed the “*4 Rs of Resilience*” to embed preventative mental health behaviours, such as help-seeking, into the virtue of ‘resilience’ commonly identified by the Irish farming community as a particular strength of Irish farmers [blinded for review]. Farmers previously reported that using “mental health” in the intervention’s title could potentially reduce attendance and suggested the use of resilience in both the title and intervention instead [blinded for review]. Based on a similar short intervention program designed to improve mental health literacy in sport (O’Keeffe et al., 2023; Sebbens et al., 2016), the “*4 Rs of Resilience*” (Table S1) guides participants through a discussion on mental health and help-seeking designed to provide foundational and accessible skills, consisting of Recognise, Reach out, Refer and Remain supportive.

**Table S1**

*The 4 Rs of Resilience with their purpose explained.*

| **Rs of Resilience** | **Purpose** |
| --- | --- |
| Recognise | Recognise symptoms and experiences of poor mental health in yourself and others |
| Reach out | Reach out to others, reach out to professional supports, and help those who reach out to you |
| Refer | Refer to a core set of skills, behaviours which support positive mental health |
| Remain Supportive | Remain supportive of yourself and others by habitualising and practising the first 3 Rs |

The intervention was underpinned by behaviour change theory (Theory of Planned Behaviour; Azjen, 1991), Self-Efficacy Theory (Bandura, 1977; Schwarzer & Fuchs, 1996), and informed by research that identified poor mental health literacy, low help-seeking intentions, and a lack of knowledge of mental health services among Irish farmers [blinded for review]. The 4 Rs of Resilience are introduced across four focused discussion topics (Table S2) designed to improve self-efficacy and encourage health behaviour change in participants who may hold resistant or negative attitudes towards mental illness or mental health concerns.**Table S2**

*Embedding theory into the ‘4 Rs of Resilience’ through four discussions*

| **Rs of Resilience** | **Content Aim** | **Theory of Planned Behaviour** | **Self-Efficacy Theory** |
| --- | --- | --- | --- |
| **Discussion 1:**  R: Recognise | Participants gain and apply knowledge about mental health literacy through discussing negative attitudes towards mental health and how to recognise signs of negative mental health in others | Address Subjective Norms that stigmatise discussion of mental illness and health by highlighting signs of ill-health  Increase Perceived Behavioural Control of engaging in helping behaviours towards others | Increase capabilities and confidence in identifying signs and symptoms of mental health and illness, especially in others |
| **Discussion 2:**  R: Recognise,  Reach out | Participants apply knowledge about other’s mental health to their own experience, improving mental health literacy and identifying how to recognise signs of negative mental health | Address Subjective Norms against vulnerability by encouraging symptom recognition.  Shift stoic attitudes by framing mental health literacy as a essential for resilience  Increase Perceived Behavioural Control around help-seeking behaviours by encouraging both reaching out to others for help or offering help to others | Empower individuals with the self-awareness necessary to take action and seek or provide mental and physical health support when needed |
| **Discussion 3:**  R: Refer (to your skills) | Participants learn next steps to symptom recognition, including accessible coping strategies and preventative mental health behaviours such as sleep, diet, exercise, and leisure | Shift subjective norms against help-seeking by including it as one of several important preventative health behaviours  Address attitudes that mental illness is inherent or unavoidable  Increase Perceived Behavioural Control of coping strategies useful in both positive and negative mental health experiences. | Increase individuals’ self-efficacy in maintaining positive mental health through daily routine, |
| **Discussion 4:**  Refer,  Remain Supportive | Participants gain knowledge of many different services available to them and others and engage with other farmers’ testimonials of successful mental health help-seeking | Address subjective norms against both help-seeking and discussing mental illness by sharing farmers’ first-hand accounts of mental distress and help-seeking.  Address attitudes that mental health help is inaccessible or unavailable by clearly outlining avenues of help-seeking  Increase Perceived Behavioural Control around utilising mental health resources by providing accessible and diverse resources. | Increase participants’ help-seeking self-efficacy through observing other farmers succeed in their help-seeking efforts |
