# Supplementary material for: “Skills for Resilience in Farming”; an evidence-based, theory driven educational intervention to increase mental health literacy and help-seeking intentions among Irish farmers
Source: PLoS One. 2025 Oct 1;20(10):e0333115. doi: 10.1371/journal.pone.0333115 (PMC12488010; doi:10.1371/journal.pone.0333115)
Supplement: S2 File — (DOCX) [file pone.0333115.s002.docx]

**Supplementary Material 2**

*Overcoming barriers reported by the Irish farming community*

**Table S3**

*Challenges to engaging farmers in mental health intervention and our approaches to addressing them (informed by research on farmer and stakeholder preferences for mental health interventions by Malone et al., (2023 [in press]).*

|  | **Challenge** | **Approach Taken** |
| --- | --- | --- |
| 1 | Reluctance to attend a mental health programme. | We delivered training through existing farm events (farm discussion groups), rather than as a standalone event. |
| 2 | Stigma associated with the title “mental health*”* | Farmers previously reported that using “mental health” in the title could deter attendance and instead suggested the use of resilience in both the title and intervention content. We used the title “*Skills for Resilience*” and focused the intervention on the *“4 Rs of Resilience*”; Recognise, Reach Out, Refer to your Skills and Remain Supportive. |
| 3 | Stigma associated with mental health help-seeking | We consistently highlighted how the overall physical and mental health of the farmer impacts the ‘wellbeing’ of the farm. We asked participants if and how the farm might be affected if a farmer was struggling. We then described the visible signs on the farm that might be present when a farmer is struggling mentally (i.e., animals not minded as well as usual, yard unkempt). In addition, we recorded two farmers’ testimonials on their mental health journeys. This aimed to 1) Allow farmers to relate to and identify with the mental health challenges of another farmer; 2) Put the theory into context for participants (i.e. poor mental health exists in farmers and farmers do seek help); 3) Educate participants on how the two farmers sought help step by step, and what supported them through it (e.g. peer support, time off-farm, hobbies); 4) The positive impact that help-seeking had on both the farm and farmer (e.g. how, since seeking help, their farms have gone from strength to strength); 5) Normalise help-seeking in farmers. In addition, we focused the intervention on how one might notice signs that *another* person was struggling. Once this was discussed and participants were comfortable in engaging, we then shifted the discussion to the farmer themselves and how they may notice if they were struggling. |
| 4 | Reluctance to engage with health professionals. | The intervention was delivered through discussion groups to increase participation, and also to educate farmers on signs of poor mental health who may be reluctant to seek help from a health professional |
| 5 | Reluctance to engage with professionals who lack farming knowledge. | [blinded for review] is from a dairy farming family and has a wealth of experience with farmers from both personal and research experiences. [blinded for review] is also an expert in mental health with training in health intervention, holding a BA Psychology and MSc in Health Psychology. During the intervention introduction, [blinded for review] highlighted both her farming background and psychology training to participants. |
| 6 | Reluctance to display mental health supports in their house/ on fridge due to perceived stigma. | We created a double sided A5 sized laminated handout with supports for participants. One side contained mental health supports, while the other side contained farm supports. This allowed participants to place the handout on their fridge/ house, with only the farm supports side visible, to minimise the stigma that may accompany the mental health support lines side. |
