# Supplementary material for: “Skills for Resilience in Farming”; an evidence-based, theory driven educational intervention to increase mental health literacy and help-seeking intentions among Irish farmers
Source: PLoS One. 2025 Oct 1;20(10):e0333115. doi: 10.1371/journal.pone.0333115 (PMC12488010; doi:10.1371/journal.pone.0333115)
Supplement: S3 File — (DOCX) [file pone.0333115.s003.docx]

**Supplementary Material 3**

*Final Intervention Script*

- Welcome. - who am I & farm background.
- When people hear “mental health”- stigma, scary but MH is the things you do every day without realising.
- [blinded for review], national study of help-seeking.
- I'm here today- chat about how we can help ourselves and others to be resilient. We’ll chat about when we notice ourselves or others struggling, how to recognise the signs, what we can do to help, and how to get professional help if needed. Throughout our discussion, I’m also going to share some results from our own chats with farmers across Ireland.
- Open discussion- want to hear your own input and expertise
- Chat for a while, we’ll have another quick survey. After this- questions.

**Discussion 1- Recognising in Others**

- We all know the importance of farming- none of us would have food without it, but farming comes with important health risks. It takes a resilient bunch to be able to do it, and that’s what we’re here to talk about. Throughout this discussion, we’re going to keep coming back to the 4 Rs of Resilience. That’s **Recognise, Reach out, Refer to your skills and Remain supportive**.
- Let’s start with the first one, Recognise. How could you recognise if someone in your **family** or **community is struggling**, what are the signs? I want to highlight a few signs to look out for.

**Mood** The first is if someone's mood changes noticeably. E.g. they may be more irritable with others than usual or seem on edge. I know that It’s easy to take these things personally if their low mood is directed at you, you could say did I do something wrong to annoy them? But these changes can actually be signs of a person dealing with their own struggles internally.

**Isolation** Similarly, the second sign is if someone is withdrawing from work, from social settings, or from their relationships. This might be their way of facing their own challenges. E.g. Maybe one of the farmers you know stops going to farm walks and shows or maybe he doesn’t turn up at his discussion group for a few weeks or months.

**The farm** Do you think there would be any signs on the farm if a farmer was struggling? Animal welfare, farm unkempt. Remember we’re just focusing on how to recognize these signs; we’ll get to what we can do about these signs once we recognise them soon.

**Discussion 2- Recognising in Yourself.**

- Now let’s shift the discussion for a second to ourselves. Recognising when someone else is struggling is different from recognising that you yourself are struggling.
- To be resilient, it’s important to be able to recognise our own health concerns, to check in with ourselves. So how can we check in with ourselves, and what are the signs we might be struggling? What signs might people recognise in themself that the people around them wouldn't?
- Main sign might be starting to **lose that passion or interest in the farm**, or you **lose interest in your hobbies**. Tight feeling in chest, dreading the day ahead, low energy. We found that 1 in 4 farmers feel like this. Another one is that you might find yourself lying awake at night, your mind is racing, and you can’t sleep. Again, we found half of farmers have poor sleep.

**Tasks:** Easiest way to recognise struggling is recognising a drop in what they can accomplish. Things that you would normally do every day, without a thought, suddenly become very draining or hard when you’re having a hard day or a hard week. You know, you’re dreading the thoughts of going out to check the animals, maybe you feel everything’s going wrong.

- Pushing through may feel like the most important thing to do to get the job done, but it’s also very important to step back and recognize that you're struggling. You know, you’d always check in on the welfare of your animals, checking their feed and water but do you check in on yourself like this?
- Part of resilience is being able to be flexible and face these challenges in different ways rather than thinking ‘sure it’ll be grand’ or ‘it’ll sort itself out’ and leaving the challenge to get bigger.
- There are a lot of things you can do to maintain and recover your health.
- We found that a lot of farmers are more likely to reach out to their family and their friends first. So, let's talk about that. Let’s say you recognise that someone in your family, or a friend or someone in your community is struggling. What can you do to help them?
- This is where we get to the second R of resilience, Reach out.
- People mightn’t reach out for help themselves. So, it really makes a difference when you **take that first step in reaching out to support them.** After you recognise that someone is struggling, the next step is to just reach out and start a conversation with them. It doesn’t have to be specific - a chat, a text, give them a ring.
- We know farmers often don’t want to talk about what they’re really struggling with immediately and that’s absolutely fine. But just listening to whatever they feel like sharing is giving them more support than you’ll ever know. And making sure they know that they don’t have to only rely on themselves.
- When people hear “talk to someone” they think this involves sitting down and telling someone “Okay look I feel anxious, stressed, down”, but to be honest it doesn’t have to be like this. Farmer told me that even just chatting to another farmer about things, let’s say the weather, or how the grass is doing, etc feels like a weightlifting off their shoulder when you talk to someone else in your shoes- they understand you
- I think discussion groups are a fantastic place for support - if you’re having a problem on the farm, there’s a high likelihood that you’re not the only one.
- Last week for e.g., I was chatting to a farmer in Galway- at a meeting a farmer stood up and said, “I need help”. By the end of the meeting, 30 out of 70 had said the same thing. Shows that you aren’t the only one, and by taking that first step to talk to someone or start a conversation has a huge impact.

**Discussion 3- How to stay healthy**

- Sometimes- a lot easier to give advice than knowing what to do when you’re in this position yourself. So, let’s talk about what you can do to keep yourself going when you’re struggling? What can you do when you're stressed or frustrated about the job? Is there anything you did in the past to help yourself through a challenging situation?
- From chatting to farmers, we found that a lot will try to toughen up when facing challenges. This is important but to be resilient it’s important to have a wide range of skills we can Refer to, that’s our next R - Refer.
- So think about these skills for resilience that we’re talking about, these Rs, as a **toolbox.** If you only have a hammer, no matter how good at using it, it’s not going to loosen a bolt. Now I know a few of you might laugh and say a little persuasion and maybe thickness would help with that, but look, the bottom line is that the job would be a lot easier with a spanner. The same applies to our health. Why would you choose to be so tough on yourself and do things the hard way, when you can use a few simple skills that would benefit your health and the farm instead?

There are a few skills you can refer to that will always help:

1. **Planning-** something so simple but highly effective. Farmers told me that writing out a list at the start of the day, or even the week puts them on track. Make a bullet point list- prioritise your time- if you know you’ve to do big tasks, why not ask for an extra pair of hands- if you ask your neighbour for a hand, chances are that they might need a hand with something else, and you can return the favour, which I’m sure some of ye already do.
2. **Sleep**- make sure you’ve got a routine that works for you. At busy times of year, you might be up at all hours of the night, so make sure that instead of sacrificing your sleep you try work in time between tasks or in the evening if you know you will be up later with let’s say a cow calving. Or even, a 10 minute nap after the dinner. I’d really recommend trying to get a good sleep- but maybe not for the next half an hour until I’m finished anyways.
3. **Good nutrition and hydration**, make sure you’re getting three meals a day and 2 litres of water. Make sure you’re not drinking too much either, from our survey, we found that 3 in 10 farmers, especially young lads, are drinking at harmful levels, which can make other tasks harder and can even lead to farm accidents. Eating well has a huge impact on your energy levels, but also your mind. Sure, you probably see it with your animals- better quality feed= higher output.
4. **Exercise:** At least 30 mins/ day, 5 days/ week. - Lowers stress, increases health.
5. **Do something you enjoy.** Make time for fun.

- Allow yourself to have some time to wind down away from the farm.
- Even going for a walk. Now look, I know you get plenty of exercise walking across the farm during the day- but the chances are during this time, you’ve 101 things on your mind- this is why it’s so important to get some time away from the farm.
- This could be maybe going to the cinema or going to watch a GAA game.
- I know it seems like you mightn’t have time but that’s why it’s so important to plan your day like I mentioned earlier- set aside some time for the things you enjoy, and I find this helps you face work a lot easier- you’re more relaxed and in a clearer headspace.
- A farmer I met last year, and I’ve stayed in touch with since, told me he takes one hour in the evening to himself. He brings the dog for a walk in his local town, but it’s 60 minutes for him to unwind after a hectic day on the farm - he said the roof could be falling in but he still makes it his business to go for the walk.

**Discussion 4 - Remain Supportive**

- The final R of resilience is to remain supportive. Maintaining our health is important and like we said earlier, when we’re struggling it can feel a lot harder. This is when having an extra pair of hands helps. Let's say someone in your community is in crisis, and you recognize this. What services can you refer them to? (GP, Counsellor, 999).
- If you had a chest infection, chances are you’d go to the GP, get antibiotics and you’d have to rest because your body wouldn’t be able to work. The exact same for your mind and mental health- if you’re feeling crap, you need to rest. You know, check in on yourself, are you getting enough good food and water? Are you getting sleep? Are you taking time away from the farm?
- Let's say you recognize that someone is struggling - not in crisis yet but struggling with their health. Showing some of these signs we’ve described. Where can they go for extra help? What services could you help refer them to?
- I think a lot of people think farmers are so independent and maybe reluctant to get help. But that’s actually not the case. We found that the majority of farmers, 75%, would reach out for support more if they could, but a lot just didn’t know how to contact local health services. And nearly 25% farmers didn’t know how to get information on mental illness, how to reach a suicide hotline, or where to go for mental health services.
- Farmers told me before they didn’t know where to get the numbers for this kind of support. So, we took this on board and put it all into a little handout for you.
- Stick this on the fridge in the house, so you’ll have it at hand if you ever need it.
- Take a picture of the handout so you can send it to someone in need. If you want to take a picture now- I can help ye do that
- We also have a website to direct someone who's looking for help to the type of help they need. If someone you know is struggling with finances, for example, it’s stressing them out, you can refer them to this website where we’ve laid out useful numbers for that person. If they want to talk to someone about debt, there’s a number there, if they need general advice on keeping their money straight, there’s a number for that. We’ve got the same for other topics that people might struggle with as well, including farmers talking about their own experience of reaching out.
- So, the whole goal of this is to have a resource for farmers who are struggling so that they can just open it up and see what kind of support they need and find something straight away. It’s not only for farmers who are struggling, you should look when you can so that you know how to help others if they need support.
- On this website we have two farmers – Sean Kelly and Shay Concannon. So these two lovely lads reached out to me and said they wanted to share their story with other farmers, to try and help them. This took a great deal of strength to be able to contact me and record their mental health story- so I really would like you to look at these two videos- they’re only about 5 minutes each and sure couldn’t you watch them while you’re eating dinner in the evening. [adapted depending if videos shown at group or not].

**Short Summary**

- To summarise, we’ve covered the 4 Rs of resilience. Recognise, Reach out, Refer and Remain supportive.
- It is important to remain supportive of others and yourself by continuing to use all these skills we’ve covered. Maintaining our health isn’t a one and done thing - you don’t get good at sport by kicking a ball around once, and the same is true for health. You keep your machinery running smoothly, your animals fed, or cows milked. Our health is exactly the same. In this case, recognising the signs you may be struggling and reaching out is part of the maintenance necessary for your health.
- I don’t think I need to get into it, but we all know of the high numbers of suicides in farmers. This is why I can’t stress how important it is to look after yourself and reach out to others.
- How many times a week do you check your animals? Now compare that to how many times you check in on yourself and how you’re feeling every week.

**Thanks for Listening and Instructions about what’s next**

- Thanks a million for your attention, participation, and your ideas.
- Any questions for me straight away, or something you were going to contribute earlier that I moved us on from?
- I’m going to hand out the second round of surveys.
- There’s a space to put your phone number as I will be following up with you in one month’s time at the next discussion group with a very brief survey to see if you found this talk useful.
- Here’s the link to the website. Just type this into google and it will come up.
