# Supplementary material for: “Skills for Resilience in Farming”; an evidence-based, theory driven educational intervention to increase mental health literacy and help-seeking intentions among Irish farmers
Source: PLoS One. 2025 Oct 1;20(10):e0333115. doi: 10.1371/journal.pone.0333115 (PMC12488010; doi:10.1371/journal.pone.0333115)
Supplement: S4 File — (DOCX) [file pone.0333115.s004.docx]

**Supplementary Material 4**

*Semi-Structured interview Guide*

**Aim 1 – Examine participants perceptions of the discussion group**

Q – Can you tell me what you thought of the discussion?

Prompts:

- Did you enjoy the discussion? If so, why (or why not)?
- Did you find the discussion beneficial? If so, why (or why not)?
- What did you think about the duration of the discussion?
- What did you think about the content of the discussion?
- What did you think about the interactive nature of the discussion?
- Did you find the discussion useful for learning more about mental health?
- Did you find the discussion useful for broaching the topic of mental health in farming?

Q – Can you tell me what you learned from the discussion (if anything)?

Prompts:

- What did you learn that you were not aware of or did not know before the discussion?
- After the discussion, do you feel that you could identify signs of struggle in another? Why? (Mood, isolation, the farm, lack of interest in farming).
- After the discussion, do you feel that you could identify signs of struggle in yourself? Why?
- What health promoting behaviours did you learn? E.g. sleep, time off farm, nutrition?
- If you or a friend were struggling now, what mental health supports are you now aware of that you would use?
- Did you find the discussion raised awareness of mental health in farmers?

Q – After participating in the discussion, do you feel more confident in helping yourself or someone you know when they are struggling?

Prompts:

- Do you feel confident that you could support someone with their mental health?
- Do you feel confident that you have the knowledge and awareness of where to send someone for help if they need it?
- Do you feel confident discussing your struggles or discussing another person’s struggles with them now after participating in the discussion?
- Do you feel confident discussing mental health now? Has participation in the discussion increased your confidence in discussing mental health?

Q – Would you recommend the discussion to other farmers?

Prompts:

Would recommend:

- Can you tell me why you would recommend the discussion?
- What did you like about the programme that would make you recommend this to other farmers?

Would not recommend:

- Can you tell me why you would not recommend the discussion?
- What did you not like about the programme that would make you recommend this to other farmers?

Q – Do you have any recommendations for what we can do to change or improve the discussion?

Prompts:

- Are there any other topics you think should be included in the discussion? If so, what?
- Is there anywhere else we could have this discussion with farmers or get this information to them, aside from discussion groups?

**Aim 2 – Examine participants perceptions of the supplementary material (handout and website)**

Q – You received a handout with information on available supports, what did you think of this?

Prompts:

- Did you find the handout with the information on available supports a useful resource? If so, why?
- What did you enjoy or like about the handout?
- What did you not enjoy or not like about the handout?
- Where will you store this handout?
- Do you think you will use this handout in future?
- Is there any additional resources or information you think should be added to this?

Q – You also received a link to an online website and a number of helpful videos, what did you think of these?

Prompts:

- Did you find the website and videos a useful resource? If so, why?
- What did you enjoy or like about the website and videos?
- What did you not enjoy or not like about the website and videos?
- How often did you view or engage with these supports?
- Will you engage with this in the future?
- Did you opt in for the 2-week reminder text message to view the website and videos? If so, did it prompt you to look at the website? Did you look at the website before you got this prompt? Why did you not look at the website before this prompt?
